# Mouse Models of Genetically Altered Peroxiredoxin 6

**DOI:** 10.3390/antiox8040077

**Published:** 2019-03-27

**Authors:** Sheldon I. Feinstein

**Affiliations:** 1 Institute for Environmental Medicine, University of Pennsylvania Perelman School of Medicine, Philadelphia, PA 19104, USA; sif@pennmedicine.upenn.edu; 2 Peroxitech, Ltd., Philadelphia, PA 19104, USA

**Keywords:** peroxidase, phospholipase A_2_, lipid peroxidation, phospholipid hydroperoxide, knockout mouse, knock-in mouse, membrane repair

## Abstract

Peroxiredoxin 6 (Prdx6) has been shown to have three enzymatic activities: peroxidase, phospholipase A_2_ (PLA_2_) and acyl transferase. The peroxidase activity is unusual, as it is capable of reducing phospholipid hydroperoxides (as well as hydrogen peroxide and short chain organic peroxides). Knockout and overexpressing mice have been produced that demonstrate the effect that eliminating or overproducing Prdx6 has on the animals’ physiology. In addition, mutations in various amino acids of Prdx6 have been identified that interfere with different enzymatic functions as well as protein transport. These mutations were originally characterized biochemically; subsequently, several knock-in mouse strains have been produced, each containing one mutation. These mice include the S32T knock-in that affects protein transport, the C47S knock-in that inactivates the peroxidase enzymatic activity, the D140A knock-in that inactivates the PLA_2_ enzymatic activity and the H26A knock-in that inactivates the peroxidase and blocks binding to phospholipids. This review summarizes the properties of these mice based upon studies conducted with the knockout, overexpressing and knock-in mice and the effect of the genetic changes on the biochemistry and physiology of these mice. The availability of these mice is also briefly discussed.

## 1. Introduction

Peroxiredoxin 6 (Prdx6) is a multifunctional enzyme with several different enzymatic activities [1,2] and has been implicated as a factor in a wide variety of diseases [3]. The molecular properties of the enzyme have been intensely studied and mutations affecting each of the activities of Prdx6 have been identified. The effects of many of these mutations, as well as the effect of deleting Prdx6 completely or of overexpressing it, have been studied in various models, including, knock-out, over-expressing and knock-in mice.

The primary purpose of this article is to review the information about Prdx6 that has emerged from studies of genetically altered mice. The mouse studies described here have, in general, been compatible with the results obtained in biochemical and in cell culture studies. However, live mice do often provide information about the role of Prdx6 that is not possible to deduce from other types of studies. 

## 2. Background: Enzymatic Activities of Prdx6 and Critical Amino Acids for Enzymatic Activities

Prdx6 has several enzymatic activities. It can reduce peroxides [4] including fatty acid peroxides and phospholipid hydroperoxides. The product of this reaction is a secondary alcohol [5]. The reaction is equally efficient for hydrogen peroxide, tert-butyl hydroperoxide, fatty acid hydroperoxides and phospholipid hydroperoxides. The only other enzyme in mammalian cells that can reduce phospholipid hydroperoxides is glutathione peroxidase 4 (GPX4), also known as phospholipid hydroperoxide glutathione peroxidase [6]. Other peroxiredoxins reduce peroxides using an active cysteine which is then partially reduced by a second conserved cysteine, forming a disulfide bond that is, in turn, restored to the thiol form by interaction with reductants thioredoxin or glutaredoxin. However, Prdx6 has a single active cysteine, at position 47 in the amino acid sequence [7,8], which is reduced by pi glutathione-s-transferase. It occurs in a consensus sequence, PV**C**TT, although the effect of mutating this consensus, other than the cysteine, on Prdx6 peroxidase activity, has not been tested. The ability to reduce phospholipid hydroperoxides [5] suggests that Prdx6 is important in the repair of damage to cell lipids, such as lipids in the cell membrane. Overexpression of Prdx6 in cells protected against membrane damage [9] while blocking expression of Prdx6 in a lung epithelial cell line increased lipid peroxidation [10]. 

Prdx6 also has a phospholipase A_2_ (PLA_2_) activity, which cleaves phospholipids at the sn2 acyl bond, releasing the headgroup, e.g., lysophosphatidylcholine from the fatty acid moiety. The preferred substrate is phosphatidylcholine (PC), followed by phophatidylethanolamine and phosphatidylglycerol. Activity is low on other substrates such as phosphatidylinositol and phosphatidyl serine [7,11,12]. The PLA_2_ catalytic activity requires a catalytic triad that includes the histidine at position 26 (H26), the serine at position 32 (S32) and the aspartate at position 140 (D140). Our studies have shown that all three residues are necessary for PLA_2_ catalytic activity and that the H26 and S32 are required for binding of the Prdx6 enzyme to its phospholipid substrate [13]. Thus, mutation of either of these two residues not only blocks the PLA_2_ activity, but also the ability to reduce phospholipid hydroperoxides. However, it has no effect on the reduction of other peroxides, both inorganic and short chain organic [13]. Since mutation of S32 or H26 gives a mixed result, mutation of D140 is the most reliable way to differentiate the effect of PLA_2_ from the other activities of Prdx6. In addition, Prdx6 has recently been shown to have acyl transferase activity [2]. This activity can synthesize dipalmitoylphosphatidylcholine (DPPC) from lysophosphatidyl choline and palmitoyl coA. Other lyso compounds such as lysophosphatidylethanolamine, lysophosphatidylglycerol, lysophosphatidylinositol and lysophosphatidylserine as well as other fatty acyl coA molecules such as stearoyl, oleoyl and arachidonoyl coAs were much less efficiently incorporated by the enzyme. Mutational analysis in our laboratory showed that mutation of the aspartate at position 31 [D31], blocks the activity. However, the acyltransferase activity is unaffected by mutations that block the other two activities. Even mutations that prevent binding to the phospholipid substrate apparently do not block the ability to bind to the lysophosphatidylcholine (LPC) substrate. The PLA_2_ and acyl transferase activities could cooperate in the repair of membrane phospholipids via the remodeling pathway in which generation of DPPC occurs via deacylation/reacylation of sn2 unsaturated PC. They are likely to also be important in the metabolism of the lipid components of pulmonary surfactant.

## 3. Prdx6 Knockout and Overexpressing Mouse Models

The Prdx6 gene is located on mouse chromosome 1 [14]. Two strains of Prdx6 null mice are available. One was produced by Wang et al. [15] and the other by our laboratory and collaborators [16]. The former mouse has a deletion in Exon III of the Pdrx6 gene, while the mouse from our laboratory has a deletion in Exon II. Both mice are anatomically normal, viable and capable of reproduction, although male null mice from our laboratory have been found to be less fertile than wild-type mice due to oxidative damage of the spermatozoa [17,18,19] as reviewed in this FORUM [20]. This has not been tested in the other Prdx6 mouse model. In general, null mice or cells derived from these mice exhibited increased sensitivity to oxidative stress, with lower survival rates, increased tissue damage and higher oxidation levels for lipids and protein [21,22,23,24]. 

Other studies with Prdx6 null mice have also been published. The Prdx6 null mice from our laboratory showed a deficiency in phospholipid catabolism, so that the mice accumulated phospholipids in their lungs as they aged. The levels of PC and disaturated phosphatidylcholine (DSPC) in the lungs (normalized to body wt) increased by about 300% in the first year of life and continued to increase thereafter. Wild- type mice had stable levels of PC and DSPC, normalized to body weight, throughout life [25]. 

A comparison of GPX1 null mice with Prdx6 null mice showed that, despite GPX1 accounting for approximately nine-fold as much GSH-dependent peroxidase activity compared to Prdx6, the Prdx6 null mice are significantly more sensitive to oxidative stress [26]. The most likely explanation is that Prdx6 can reduce phospholipid hydroperoxides while GPX1 cannot. In fact, lungs from Prdx6 null mice do not exhibit any detectable enzymatic activity for reduction of phospholipid hydroperoxides, suggesting that the lungs of these mice do not contain GPX4; this was confirmed on a Western blot showing relatively abundant GPX4 in testis, but nothing in lung [26]. 

We have also found [27] that the PLA_2_ activity of Prdx6 triggers superoxide production by activating the reduced form of nicotinamide adenine dinucleotide phosphate (NADPH) oxidase 2 (Nox2) so that the Prdx6 null mice do not activate Nox2. This lack of Nox2 activation might be expected to result in reduced damage to tissues of Prdx6 null mice in some conditions associated with increased oxidant stress, which would mitigate their reduced repair capacity, due to a lack of Prdx6. 

Our laboratory also used the Prdx6 null mice as a source for cells that do not have Prdx6. Mouse pulmonary microvascular endothelial cells (MPMVECs) were obtained from the null mice and used in transfection or infection studies in which various expression constructs of wild-type or mutant Prdx6 could be introduced into the cells and their effects studied. These were used to show the role of Prdx6 in superoxide generation [27] and in protection of cells against lipid peroxidation [24]. 

A Prdx6 overexpressing mouse model was produced by introducing a mouse Prdx6 gene, as a transgene, into wild-type mice. Mice with multiple copies of the gene were analyzed and a line was chosen whose expression level of Prdx6 was more than an order of magnitude higher in the aorta than the wild-type mice [28]. These mice were subsequently shown to have increased resistance to oxygen toxicity as compared to wild-type mice [29] and also an increased turnover rate for lung DPPC, supporting a role for Prdx6 in surfactant metabolism [30]. 

## 4. Mouse Knock-in Models for Prdx6 Mutations

Knock-in mice are transgenic mice in which a gene of interest has been replaced by a copy of the same gene with a different sequence. The change can be as small as a mutation consisting of a single nucleotide difference. The knock-in mice described here were prepared by the method known as “recombineering” which relies on cloning using homologous recombination, generally performed in *E. coli* [31]. Initially, the mutation is introduced into a cloned copy of the mouse gene in a plasmid that contains a neomycin resistance cassette flanked by two flippase recombinase targetFRT sites. Subsequently, the mutation is introduced into mouse embryonic stem (ES) cells from the mouse strain of interest by selecting for neomycin resistance. The neomycin resistant cells are mixed into mouse embryos from mice with a different coat color. Mice born from these chimeric embryos that exhibited evidence of the coat color contributed by the embryonic stem cells are tested for germline transmission of the coat color and subsequently, the mutation. Mutant mice are bred to homozygosity and the neomycin cassette can be removed by mating with mice containing the Flippase gene which is itself removed by further breeding. 

It should be noted that the generation of the mice described here was completed several years ago; today, it is possible to generate mice much more rapidly and easily using clustered regularly interspaced short palindromic repeats (CRISPR)/CRISPR associated protein 9 (Cas9) technology in which rodent embryos can be electroporated directly, eliminating the need for using ES cells [32]. 

The knock-in mice described below were generated by the Gene Targeting Core and Laboratory and the Transgenic and Chimeric Mouse Facility of the University of Pennsylvania (Philadelphia, PA, USA). 

### 4.1. Directing Prdx6 to Lamellar Bodies and Lysosomes: The S32T Mutation

A portion of Prdx6 protein in the lung is found in lamellar bodies and lysosomes; presumably, it is also found in lysosomes in other organs as well although that has not been evaluated. Our laboratory set out to study the mechanism for this targeting. Deletion analysis was performed on a Prdx6 mammalian expression plasmid that was fused with a gene coding for green fluorescent protein GFP on the N-terminus. The mutant plasmids were transfected into cell lines and the GFP fluorescence was examined for co-localization with a stain for lamellar bodies (Nile Red) or lysosomes (Lysotracker Red). The results of these studies identified a region of Prdx6, amino acids 31–40, that was responsible for the targeting. This region includes the Serine 32 that is the part of the catalytic site of the PLA_2_ activity. Site-directed mutagenesis studies showed that mutating the Serine 32 to Alanine or the Glycine 34 to Leucine prevented the targeting [33]. Subsequently, it was shown that the chaperone protein 14-3-3-epsilon, after its activation by mitogen-activated protein kinases (MAPK kinases): ERK or P38, plays a role in the targeting process [34]. 

Further studies indicated that, unlike the Serine 32 to Alanine (S32A) mutation, the Serine 32 to Threonine (S32T) mutation did not abolish the PLA_2_ activity of the Prdx6 in recombinant protein produced in *E. coli* nor in recombinant protein produced in mammalian cells by infection with lentiviral constructs. The S32A mutation also prevented the Prdx6 from binding to phospholipids, however, this was not the case for the S32T mutation. Thus, in contrast to the S32A mutation the S32T mutation did not interfere with the ability of the Prdx6 to cleave phospholipids. However, mammalian cells did not transport the S32T mutant protein to lamellar bodies as they did for wild-type [35]. 

A knock-in mouse was constructed in which the Serine 32 was mutated to threonine. Studies with these mice showed that the mutation had no effect on the PLA_2_ enzymatic activity of the protein. However, unlike the wild-type, the Prdx6 was not transported to lamellar bodies. This was apparent both using immunohistochemical studies and also analysis of purified lamellar body fractions. Studies showed that the 14-3-3-Epsilon chaperone protein could not bind to the S32T protein, suggesting a possible mechanism for the defect in transport [35]. Thus, the data from the S32T mouse indicates that T can substitute for S at position 32 for preservation of the physical structure of Prdx6 and the ability of the enzyme to bind to and cleave phospholipids, but not for the trafficking of the protein to lamellar bodies. 

### 4.2. Abolishing the Ability of Prdx6 to Reduce Peroxides: The C47S Mutation

Studies on recombinant protein had indicated that the phospholipase A_2_ activity and the phospholipid reductase activity were located in different parts of the protein and had different active centers. The cysteine at position 47 in the amino acid sequence of Prdx6 was shown to be the active site for the reduction of the phospholipid hydroperoxide. This cysteine (shown in bold) occurs in a consensus amino acid sequence for peroxidase activity, PV**C**TT. Mutating this cysteine to serine abolished the ability of the protein to reduce any hydroperoxide. However, this mutation had no effect on the PLA_2_ activity of the Prdx6 enzyme [7] except that it did prevent the increase in PLA_2_ activity associated with oxidation of the Prdx6 protein [36,37]. Plasmids containing the Prdx6 with the C47S mutation were able to restore PLA_2_ activity, but not peroxidase activity, when transfected into Prdx6 null cells. Nevertheless, these mutant plasmids were able to partially rescue the ability of the cells to recover from oxidative stress [24]. We developed knock-in mice containing the C47S mutation [38]. When the lungs of these mice were isolated and analyzed biochemically, there was no GSH-dependent lipid peroxidase activity, indicating that GPx4 (phospholipid hydroperoxide GSH peroxidase), the other major enzyme capable of reducing phospholipid hydroperoxides [6], is not active in mouse lungs. The recovery of the C47S mouse lungs from oxidative stress brought on by hyperoxia in vivo, monitored by reduction of membrane lipids, as indicated by quantitative assays of lipid peroxide, was slower than the phospholipid hydroperoxide reduction of wild-type mice, but much better than that seen for Prdx6 null mice. Similar results were obtained with perfused lungs from the mutant mice that had been treated with tert-butylhydroperoxide to induce oxidative stress, except that the recovery time was much shorter (about ten-fold less ex vivo than in vivo) [38]. This data was also in agreement with rescue experiments in cells [24]. Since the Prdx6 null mice could not recover, but the C47S mutant mice did recover, although more slowly, the data suggested that there is another pathway for cells and lungs to recover from lipid peroxidation brought on by oxidative stress. This pathway is likely to involve the coupled phospholipase A_2_/LPCAT activities of Prdx6 which could bring about repair by the deacylation–reacylation pathway.

### 4.3. Abolishing the Phospholipase A_2_ Activity of Prdx6: The D140A Mutation

The catalytic residue for the PLA_2_ activity of the Prdx6 enzyme is the serine at position 32. This serine residue is part of a catalytic triad that also includes the histidine at position 26 and the aspartate at position 140. In the higher order structure of the enzyme, these residues are in proximity to each other and appear to cooperate to form the catalytic triad. Since our data showed that the H26 and the S32 are important for the Prdx6 enzyme to bind to phospholipids but the D140 residue is not, we used a mutation of the aspartate at position 140 to alanine (D140A) to construct a knock-in mouse that lacked the PLA_2_ activity of Prdx6 [38]. 

As predicted, lung extracts from these mice had no PLA_2_ activity. The peroxidase activity was unaffected even on phospholipid substrates, indicating that binding to phospholipids was not inhibited. Nevertheless, in experiments examining recovery from injury brought on by oxidative stress, in perfused lung and live mice [38], the data clearly show that the loss of PLA_2_ activity has a marked effect on recovery, although the effect was slightly less than that of the C47S mutation. This result is in agreement with data that we had obtained previously looking at the ability of a plasmid expressing D140A mutant protein to rescue Prdx6 null cells from damage caused by oxidative stress [24]. Thus, repair of the damage to lungs and lung cells resulting from oxidative stress is repaired exclusively by Prdx6 and can occur by either of two pathways that complement each other: by reducing the oxidized molecules or by excision and repair. The data indicate that the reduction pathway seems slightly more efficient, but that the two pathways are almost equally effective and that both are needed for maximal efficiency. 

### 4.4. Abolishing Both the Phospholipase A_2_ Activity of Prdx6 and the Ability of Prdx6 to Reduce Phospholipids: The H26A Mutation

In contrast to the D140 residue, which is necessary for the phospholipase A_2_ catalytic activity, the other two members of the PLA_2_ catalytic triad, H26 and S32, are also required for Prdx6 binding to phospholipids and thus for reduction of phospholipid hydroperoxides. Therefore, the S32A mutants and the H26A mutants would not be expected to have the acidic, calcium-independent PLA_2_ activity (aiPLA_2_) nor the ability to repair oxidized phospholipids in membranes, but would still be able to reduce short chain peroxides. It should be noted that some laboratories have used the S32A mutation to inactivate the Prdx6 PLA_2_, apparently without realizing that they are also affecting the peroxidase activity by preventing the enzyme from reducing phospholipids. For example, Ho et al. [39] concluded that the PLA_2_ activity of Prdx6 is necessary for lung cancer cell invasion, by showing that Prdx6 carrying the S32A mutation was much less stimulatory of the invasiveness of transfected A549 cells than wild-type Prdx6 and concluded that this was due to the lack of PLA_2_ activity. In fairness, the study also showed that C47S mutation did not have this effect, indicating that the lack of invasiveness was not due to the loss of peroxidase activity. However, in another study looking at cell proliferation, in one cell line (Mel-Ho) the authors conclude that both the peroxidase and PLA_2_ activities of Prdx6 are necessary for proliferation because neither S32A nor C47S mutant Prdx6 could rescue the decrease in cell proliferation caused by siRNA to Prdx6 [40]. In that case, the inability to restore proliferation could be due to the lack of ability to reduce phospholipid hydroperoxides, as both mutations affect this. 

H26A knock-in mice were produced [2] and, as expected, lung homogenate from these mice exhibited very low levels of aiPLA_2_ activity and lacked the ability to reduce phospholipid hydroperoxides, however, the ability to reduce short chain hydroperoxides or hydrogen peroxide was only about 10% below wild-type. Thus, the H26A mutation would be useful in determining the importance of the ability to reduce short chain peroxides in the overall ability of Prdx6 to protect against oxidative damage. The time course of the ability of these H26A knock-in mice to repair damage was compared with wild-type and knockout mice. In one series of experiments, whole mice were treated with hyperoxia, while in another set of experiments, perfused lungs from the mice were treated with short-chain hydroperoxide [41]. In both experimental sets, the H26A mutation was similar to the Prdx6 knockout in its inability to protect against oxidative stress, indicating that the ability of Prdx6 to reduce inorganic and short chain peroxides, which is retained by the H26A mice, is not enough to confer protection. The results of these experiments also matched experiments in which an H26A mutant construct in a lentiviral vector was introduced into pulmonary microvascular cells from Prdx6 null mice, by infection, and compared with cells infected with wild-type construct or vector alone [41]. It is not surprising that the H26A mutation cannot repair the damage to the lungs as this requires either the ability to reduce or cleave phospholipid hydroperoxides and the H26A mutation is unable to fulfill either function. 

## 5. Conclusions and Future Directions

Prdx6 is a complicated enzyme, implicated in many physiological processes and diseases, as reviewed in this FORUM [42]. Our understanding of the biochemistry of Prdx6 has been greatly facilitated by mutation of constructs for Prdx6 expression and the examination of the effects of the mutations on enzyme function. However, in order to understand the physiological effects of these mutations, it is necessary to place them in a living organism. We have used genetically altered mice to explore the effects of deleting Prdx6, overexpressing it or mutating it on mouse physiology and metabolism. The results have been helpful in furthering our understanding of the role of Prdx6 and have also shown that our in vitro and cell culture studies give us useful information that is generally borne out by the studies in mice. A summary of the mice described in this review is shown in Table 1. 

The mice described here should turn out to be useful in studies of many diseases, as Prdx6 has already been linked to quite a number of them [3]. For example, studies of male fertility have already pointed to the involvement of Prdx6 [18,19,20,21]. We have archived all of our mice described here to be available from the MMRRC Repository. Our archived knock-in mice have had the neomycin resistant cassette removed and the Flippase gene bred out. The other Prdx6 knockout mouse and the Prdx6 overexpressing mouse are available from the Jackson Laboratory, Bar Harbor, ME, USA. 

Our studies showing that the S32T mouse does not transport Prdx6 to lamellar bodies [33] confirm our cell culture studies in which we identified the region of Prdx6 involved in targeting [33]. It is also notable that mutating the serine 32 to a threonine preserves enzyme activity but blocks the transport of the protein [35]. More studies will be needed to determine if the loss of Prdx6 from lamellar bodies has any effect on resistance to oxidative stress and whether the effect of this mutation is similar to the knockout mouse in causing the accumulation of phospholipids in the lung. 

Our studies on the D140A and C47S mice have involved mostly repair of oxidative damage. Before these experiments were performed, it seemed likely that the peroxidase activity would be much more important in repair than the PLA_2_, since it seemed to be a more efficient method of repair. However, it turned out that although the peroxidase activity is more important, the difference is slight and both activities are needed for wild-type repair efficiency [38]. Again, these studies confirmed what had been found in cell culture [24]. 

The Prdx6 null mouse was much more sensitive to oxidative stress than the GPX1 null mouse, even though there is much more GPX1 protein and activity in the lung than Prdx6 [26]. Our studies with the H26A knock-in mouse [41] support our hypothesis that the reason for this is that Prdx6 can reduce phospholipid hydroperoxides, while GPX1 cannot. The H26A Prdx6 cannot bind to phospholipids and the mouse expressing this mutation is no more protected from oxidative stress than the Prdx6 null mouse. 

There are some other potential knock-in mice containing other mutations of Prdx6 that would be useful, however, they have not yet been produced. The improved technology available today would facilitate the production of these mice. It will be interesting to test whether the recently discovered lysophosphatidyl choline acyl transferase activity [2] combines with the PLA_2_ activity to perform the repair of membrane phospholipids; these studies would be facilitated by the creation of a knock-in mouse for the D31A mutation which inactivates the acyl transferase. A knock-in mouse model of this mutation would also be useful in evaluating the role of the acyl transferase in the metabolism of pulmonary surfactant. 

Prdx6 is phosphorylated by MAP kinases, resulting in an increase in PLA_2_ activity of more than an order of magnitude and a broadening of the pH optimum of the enzyme so that activity at pH 4 and pH 7.4 is roughly equivalent [43]. However, phosphorylation has no effect on the peroxidase activity. Phosphorylation has been found to take place on the threonine 177 (T177) of the Prdx6 protein. Mutation of the threonine to an alanine (T177A) abolishes the phosphorylation and the increase in activity. Mutation of the threonine to a glutamic acid (T177E) also prevents phosphorylation, but increases the PLA_2_ activity about two-fold, presumably because the glutamate mimics the charge of the phosphate group (43]. Since the PLA_2_ activity of Prdx6 plays roles in surfactant metabolism, membrane repair and activating NADPH oxidase 2 to produce superoxide, it would be interesting to generate T177A (and possibly T177E) knock-in mice and to study the effect of manipulating Prdx6 phosphorylation on these processes. 

The active cysteine in Prdx6 is regenerated through heterodimerization with glutathione-S-transferase pi. Mutations at leucine 145 (L145) or leucine 148 (L148] interfere with the heterodimerization and the ability of the Prdx6 to reduce phospholipid hydroperoxides. However, there is no effect on PLA_2_ activity. The double mutation (L145A; L148A) essentially eliminates the heterodimerization and the peroxidase activity of the enzyme in vitro [44]. A knock-in mouse model containing the double mutation (L145A; L148A) would be expected to have a phenotype similar to the C47S, although perhaps less severe and this could be tested in models of oxidative stress. 

The role of Prdx6 in physiology and disease is complex because of its multiple activities. In some cases, one or another of the enzyme activities has been suggested as a factor in a particular disease. The availability of mouse models in which the effect of the absence of each activity can be studied independently will assist in identifying the role of these mutations in normal physiology and in pathology and may point towards possible targets for drugs directed against these diseases. 

## Figures and Tables

**Table 1:** Summary of the description of mice discussed in this review. PLA_2_: Phospholipase A_2_; MMRRC: Mutant Mouse Resource and Research Center.

Description of Prdx6 in Mouse	References	Prdx6 Peroxidase Activity Against Inorganic and Short-Chain Peroxides	Prdx6 Peroxidase Activity Against Phospholipid Hydroperoxides	PLA_2_ Activity	Transport to Lamellar Bodies	Mice Available from:
Knockout (1)	[15]	No	No	No	N/A	Jackson Labs
Knockout (2)	[16,17,18,19,20,21,22,23,24,25,26,27]	No	No	No	N/A	MMRRC
Overexpressing	[28,29,30]	Yes	Yes	Yes	Yes	Jackson Labs
S32T	[35]	Yes	Yes	Yes	Yes	MMRRC
C47S	[38]	No	No	Yes	No	MMRRC
D140A	[38]	Yes	Yes	No	Yes	MMRRC
H26A	[41]	Yes	No	No	Yes	MMRRC

N/A: Not applicable.
